# Supplementary material for: Square-root higher-order Weyl semimetals
Source: Nat Commun. 2022 Sep 24;13:5601. doi: 10.1038/s41467-022-33306-9 (PMC9509390; doi:10.1038/s41467-022-33306-9)
Supplement: Supplementary file 1 — Supplementary Information [file 41467_2022_33306_MOESM1_ESM.pdf]

Supplementary Information

Square-root higher-order Weyl semimetals

Lingling Song, Huanhuan Yang, Yunshan Cao, and Peng Yan

School of Electronic Science and Engineering and State Key Laboratory of Electronic Thin Films and Integrated Devices, University of Electronic Science and Technology of China, Chengdu 610054, China.

SUPPLEMENTARY NOTE 1. THE SQUARED HAMILTONIAN

It is noted that $\mathcal{H}$ [Eq. (2) in the main text] is chiral-symmetric, because it meets the condition $\mathcal{H} = -\gamma\mathcal{H}\gamma$ with

$$\gamma = \begin{pmatrix} I_{2,2} & O_{2,3} \\ O_{3,2} & -I_{3,3} \end{pmatrix}, \tag{1}$$

where $O_{2,3}$ ($I_{2,2}$) and $O_{3,2}$ ($I_{3,3}$) are the 2×3 (2×2) and 3×2 (3×3) zero (identity) matrices, respectively. The Hamiltonian $\mathcal{H}$ with chiral symmetry indicates the existence of a parent Hamiltonian $\mathcal{H}^2$. With the help of parent Hamiltonian, one can obtain the eigenvalues of $\mathcal{H}$ by taking its square

$$\mathcal{H}^2 = \begin{pmatrix} h^H_{\mathbf{k}} & O_{2,3} \\ O_{3,2} & h^K_{\mathbf{k}} \end{pmatrix}, \tag{2}$$

where $h^H_{\mathbf{k}} = \Phi^\dagger_{\mathbf{k}}\Phi_{\mathbf{k}}$ and $h^K_{\mathbf{k}} = \Phi_{\mathbf{k}}\Phi^\dagger_{\mathbf{k}}$ represent the Hamiltonian of a stacked honeycomb sublattice and breathing kagome sublattice, respectively. Their explicit expressions are

$$h^H_{\mathbf{k}} = \begin{pmatrix} h_{11} & h_{12} \\ h^*_{12} & h_{22} \end{pmatrix}, \tag{3}$$

with

$$\begin{aligned} h_{11} &= 3t_a^2, \\ h_{12} &= t_at_b + 2t_at_z\cos k_z + (t_at_b + 2t_at_z\cos k_z)e^{-i\mathbf{k}\cdot\mathbf{a}_1} + (t_at_b + 2t_at_z\cos k_z)e^{-i\mathbf{k}\cdot\mathbf{a}_2}, \\ h_{22} &= 3t_b^2 + 12t_bt_z\cos k_z + 6t_z^2 + 6t_z^2\cos(2k_z), \end{aligned} \tag{4}$$

and

$$h^K_{\mathbf{k}} = \begin{pmatrix} h_{33} & h_{34} & h_{35} \\ h^*_{34} & h_{44} & h_{45} \\ h^*_{35} & h^*_{45} & h_{55} \end{pmatrix}, \tag{5}$$

with

$$\begin{aligned} h_{33} = h_{44} = h_{55} &= t_a^2 + t_b^2 + 2t_z^2 + 4t_bt_z\cos k_z + 2t_z^2\cos(2k_z), \\ h_{34} &= t_a^2 + [t_b^2 + 2t_z^2 + 4t_bt_z\cos k_z + 2t_z^2\cos(2k_z)]e^{i\mathbf{k}\cdot\mathbf{a}_1}, \\ h_{35} &= t_a^2 + [t_b^2 + 2t_z^2 + 4t_bt_z\cos k_z + 2t_z^2\cos(2k_z)]e^{i\mathbf{k}\cdot\mathbf{a}_2}, \\ h_{45} &= t_a^2 + [t_b^2 + 2t_z^2 + 4t_bt_z\cos k_z + 2t_z^2\cos(2k_z)]e^{-i\mathbf{k}\cdot(\mathbf{a}_1-\mathbf{a}_2)}. \end{aligned} \tag{6}$$

We note that $h^H_{\mathbf{k}}$ and $h^K_{\mathbf{k}}$ have the same energy band solution, except that $h^K_{\mathbf{k}}$ has an additional flat band pinned to zero energy. The energy band solution of the $h^K_{\mathbf{k}}$ is

$$E_{\mathbf{k}} = 0 \ \text{ and } \ \frac{3}{2}\left[t_a^2 + t_b'^2 \pm \sqrt{(t_a^2 - t_b'^2)^2 + 4t_a^2t_b'^2|\Delta(\mathbf{k})|^2}\right], \tag{7}$$

with $t_b' = t_b + 2t_z\cos(k_z)$ and $\Delta(\mathbf{k}) = (1 + e^{i\mathbf{k}\cdot\mathbf{a}_1} + e^{i\mathbf{k}\cdot\mathbf{a}_2})/3$. The band structure of the original Hamiltonian is therefore given by $\varepsilon_{\mathbf{k}} = \pm\sqrt{E_{\mathbf{k}}}$.

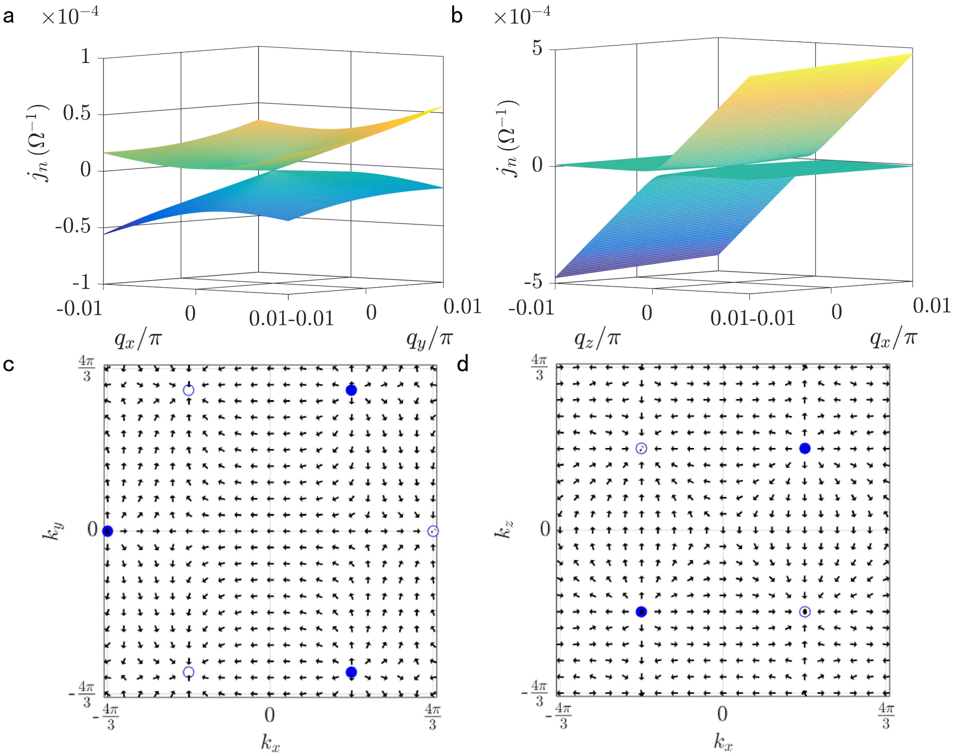

Supplementary Figure 1. The admittance dispersion around the Weyl point K_+ in the **a** $q_x - q_y$ and **b** $q_x - q_z$ planes. Here we are particularly interested in the 1st band, because the Weyl points appear at the intersection between the first and second energy bands. The spatial distribution of the Berry curvature for the 1st band around **c** $k_x - k_y$ ($k_z = k_{zw}$) and **d** $k_x - k_z$ ($k_y = 0$) planes. Open and solid circles represent the Weyl points with opposite topological charges +1 and −1.

SUPPLEMENTARY NOTE 2. THE LINEAR ADMITTANCE SPECTRUM NEAR THE WEYL POINT AND THE BERRY CURVATURE

In this section, we demonstrate that the Weyl semimetal in our system hosts linear dispersion in all three dimensions in the vicinity of the Weyl points which act like monopoles of Berry curvature. To this end, we expand $h^H_{\mathbf{k}}$ in terms of Pauli matrix $h^H_{\mathbf{k}} = \lambda_0\sigma_0 + \lambda_x\sigma_x + \lambda_y\sigma_y + \lambda_z\sigma_z$ with σ_0 the identity matrix, σ_x, σ_y and σ_z being the Pauli matrices. The parameters λ_i ($i = 0, x, y, z$) are explicitly expressed as

$$\begin{aligned}
\lambda_0 &= \frac{3}{2}t_a^2 + \frac{3}{2}t_b^2 + 6t_bt_z\cos k_z + 3t_z^2 + 3t_z^2\cos(2k_z),\\
\lambda_x &= t_at_b + 2t_at_z\cos k_z + 2(t_at_b + 2t_at_z\cos k_z)\cos(\frac{1}{2}k_x)\cos(\frac{\sqrt{3}}{2}k_y),\\
\lambda_y &= 2t_at_b\cos(\frac{1}{2}k_x)\sin(\frac{\sqrt{3}}{2}k_y) + 4t_at_z\cos(\frac{1}{2}k_x)\sin(\frac{\sqrt{3}}{2}k_y)\cos k_z,\\
\lambda_z &= \frac{3}{2}t_a^2 - \frac{3}{2}t_b^2 - 6t_bt_z\cos k_z - 3t_z^2 - 3t_z^2\cos(2k_z).
\end{aligned} \tag{8}$$

Near the point $\mathbf{K}_+ = (4\pi/3, 0, k_{zw})$, using the Taylor expansion, the parameters λ_i $(i = 0, x, y, z)$ of the effective Hamiltonian can written as:

$$\begin{aligned}
\lambda_0 &= \frac{3}{2}t_a^2 + \frac{3}{2}t_b^2 + \frac{3}{2}t_z^2 + \frac{3}{2}t_b t_z - 3\sqrt{3}(t_b t_z - t_z^2)q_z, \\
\lambda_x &= t_a t_z - \frac{\sqrt{3}}{2}(t_a t_b - t_a t_z)q_x - \sqrt{3}t_a t_z q_z, \\
\lambda_y &= -\frac{\sqrt{3}}{2}(t_a t_b - t_a t_z)q_y, \\
\lambda_z &= \frac{3}{2}t_a^2 + \frac{3}{2}t_b^2 - \frac{3}{2}t_z^2 - \frac{3}{2}t_b t_z + 3\sqrt{3}(t_b t_z - t_z^2)q_z,
\end{aligned} \tag{9}$$

with $\mathbf{q} = \mathbf{k} - \mathbf{K}_+$. From Supplementary Eqs. (9), one can clearly see that the band linearly touches at K_+, which is a typical feature of band crossing of Weyl semimetals. The energy bands around the Weyl point of SHOWS inherited from Supplementary Eqs. (9) are also linear. We then investigate the distribution of Berry curvature in momentum based on the low-energy effective Hamiltonian expanding around the Weyl points. It will be demonstrated that the Weyl points will generate Fermi arc states on the surface. We first consider the degenerate point at K_+. Here, we plot the 3D band dispersion around K_+ in Supplementary Figures **1a**, **b**. The band dispersion around K_- is similar to the case around K_+. Obviously, the band dispersion around the degenerate points along any direction is linear. Furthermore, the Berry curvature is expressed as

$$F_x = \frac{\partial A_z}{\partial q_y} - \frac{\partial A_y}{\partial q_z}, F_y = \frac{\partial A_x}{\partial q_z} - \frac{\partial A_z}{\partial q_x}, F_z = \frac{\partial A_y}{\partial q_x} - \frac{\partial A_x}{\partial q_y}, \tag{10}$$

where $A_\mu = -i\langle\phi|\nabla_\mu|\phi\rangle$ is the berry connection, with $\mu = x, y, z$ and $\phi(\mathbf{q})$ being its wave function. Supplementary Figures **1 c**, **d** show that the flux of the Berry curvature flowing from K_+ to K_-, which is similar to the magnetic monopole in momentum space. The monopole charge is defined as

$$C_{\mathrm{FS}} = \frac{1}{2\pi}\oint\!\!\oint_{\mathrm{FS}} \mathbf{F}(\mathbf{k}) \cdot d\mathbf{S}, \tag{11}$$

where FS is the curved surface surrounding the Weyl point. By evaluating C_{FS}, we find that K_+ and K_- are a pair of Weyl points with opposite charge $+1$ and -1, denoted by the open and solid circles respectively. This means that this 3D circuit system hosts four Weyl points that reside at the same admittance and is thus a Weyl semimetal.

SUPPLEMENTARY NOTE 3. THE 3D SQUARE-ROOT HOTI

The non-zero bulk polarization (in Supplementary Figure **2c**) gives rise to the hinge states in a triangular prism sample with the dispersion connecting the projections of the Weyl points along the k_z direction, as shown by the hinge state distribution in Supplementary Figure **2d**.

It is worth mentioning that a 3D square-root HOTI can also emerge in our system for other parameters (see Supplementary Figures **2e-g**). Comparing the bulk band structures with Supplementary Figure **2b**, one can see that the band gap of high-order topological insulators always exists from K to $\bar{K}$ (see Supplementary Figure **2e**). In this region, the bulk polarization is always non-zero in Supplementary Figure **2f**, but not the case for Supplementary Figure **2c**. For the surface band in Supplementary Figure **3a**, the Zak phase is written as

$$\mathcal{Z}(k_z) = i\int_0^{2\pi} u^\dagger(k_x, k_z)\nabla_{k_x} u(k_x, k_z)dk_x (\mathrm{mod}\ 2\pi), \tag{12}$$

with $u(k_x, k_z)$ the wave functions of the surface states. We calculate the edge topological invariant [2, 3] and find exactly the same phase transition point at $\pm k_{zw}$, as shown in Supplementary Figure **3b**. Although it is not our purpose to resolve the debate between the edge invariant and bulk invariant to characterize the topology of hinge states, we sincerely show that these two approaches lead to the same prediction of the emergence of the hinge state in our lattice structure.

SUPPLEMENTARY NOTE 4. CALCULATIONS OF THE FERMI ARCS

The Fermi arc is the equi-energy contour of the surface states at a fixed $j_n = 0.004082\ \Omega^{-1}$. Supplementary Figure **4a** shows the Fermi arcs with the same energy of Weyl points. Because all the four Weyl points are at the same

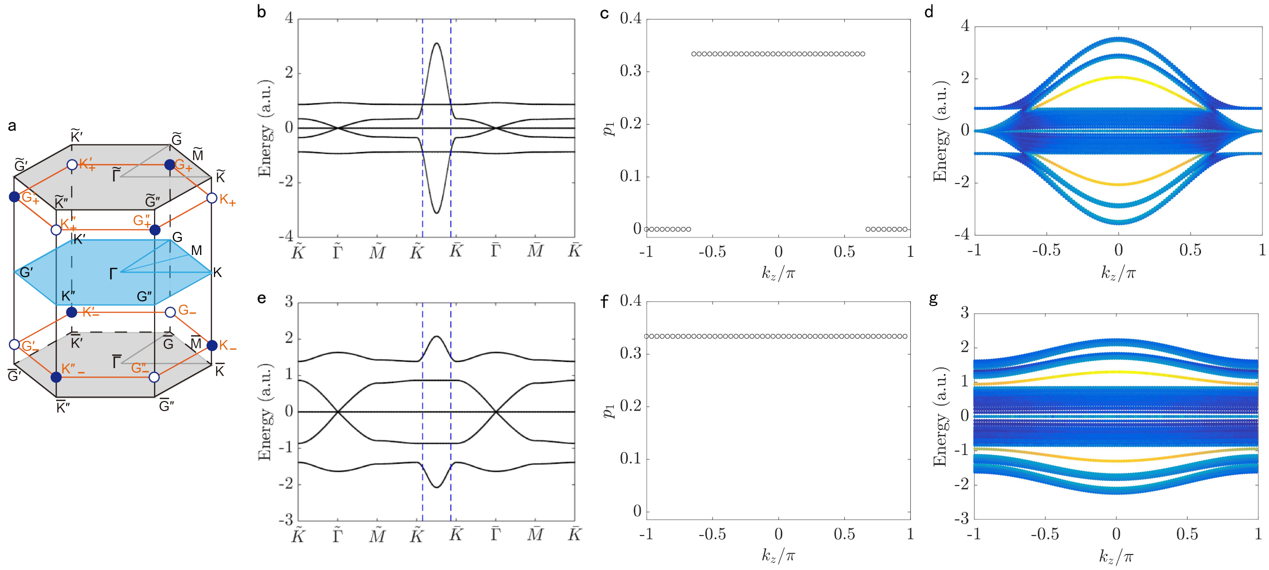

Supplementary Figure 2. **a** The first Brillouin zone and the distribution of the Weyl points. The parameters in **b**, **c**, and **d** were chosen as with $t_a = 0.5$, $t_b = 1$, and $t_z = 0.5$, corresponding to SHOWS. As a comparison, the parameters in **e**, **f** and **g** were chosen as with $t_a = 0.5$, $t_b = 1$, and $t_z = 0.1$, corresponding to square-root HOTI. **be** Bulk band structures. Here, $\tilde{K}$ ($\tilde{\Gamma}$, $\tilde{M}$) and $\bar{K}$ ($\bar{\Gamma}$, $\bar{M}$) correspond to the up and down translation of π from K (Γ, M), respectively. **cf** Bulk polarization p_1 as a function of k_z, with the subscript 1 indicating the 1st band. **dg** The projected dispersion of a triangular prism, i.e., admittance along the k_z direction. The yellow line segments indicate the hinge state dispersion.

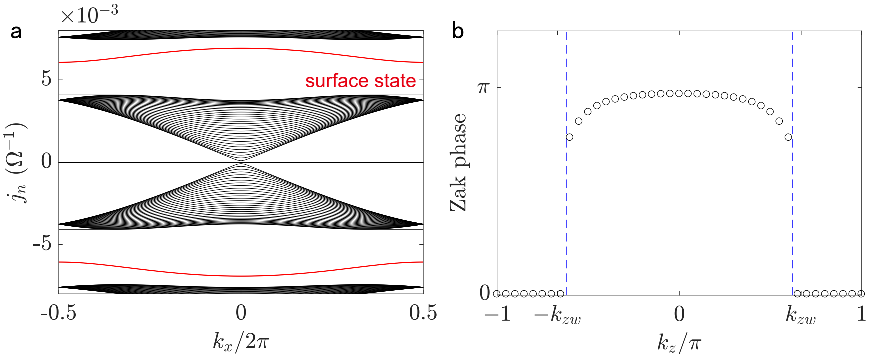

Supplementary Figure 3. **a** Dispersions of the slab geometry for $k_z = 1.66$. The red line denotes the surface state. **b** The topological edge invariant (Zak phase) as a function of k_z.

energy, the Fermi arcs connect two Weyl points with opposite charges. These surface states are clearly gapped, as shown in Supplementary Figures **4b-f**. We analyze the Fermi arcs at f=835 kHz of Weyl points in frequency space. Supplementary Figure **5a** shows the Fermi arcs with the same frequency of Weyl points. These surface states are clearly gapped, as shown in Supplementary Figures **5b-f**.

SUPPLEMENTARY NOTE 5. MAPPING FROM KIRCHHOFF'S LAW TO SCHRÖDINGER EQUATION

We derive the relation between Kirchhoff's laws and Schroedinger equation, which enables us to calculate the frequency spectrum.

In electric circuits, the equation of motion is given by

$$\frac{d\mathbf{I}(t)}{dt} = C\frac{d^2\mathbf{V}(t)}{dt^2} + L\mathbf{V}(t), \tag{13}$$

where $\mathbf{V}$ is the N-component voltage measured at each node against the ground and $\mathbf{I}$ is the N-component input current at each node.

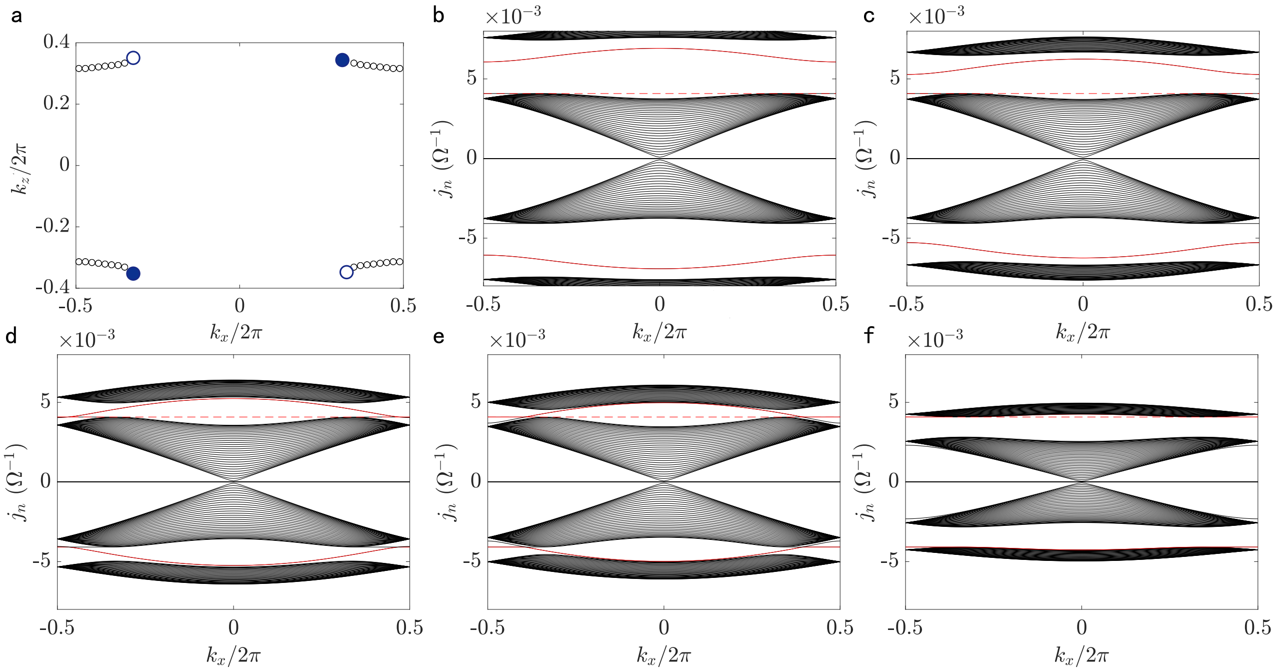

Supplementary Figure 4. **Fermi arc and dispersions of the slab geometry.** **a** The contour of the surface states at the admittance of the Weyl points ($j_n = 0.004082\ \Omega^{-1}$). The open and solid circles denote the Weyl points with opposite topological charges. **b-f** Dispersions of the slab geometry along the k_x direction for different k_z (1.66, 1.78, 1.975, 2.03, and 2.283). The solid red line denotes the surface state dispersion and the dashed line shows the position of $j_n = 0.004082\ \Omega^{-1}$.

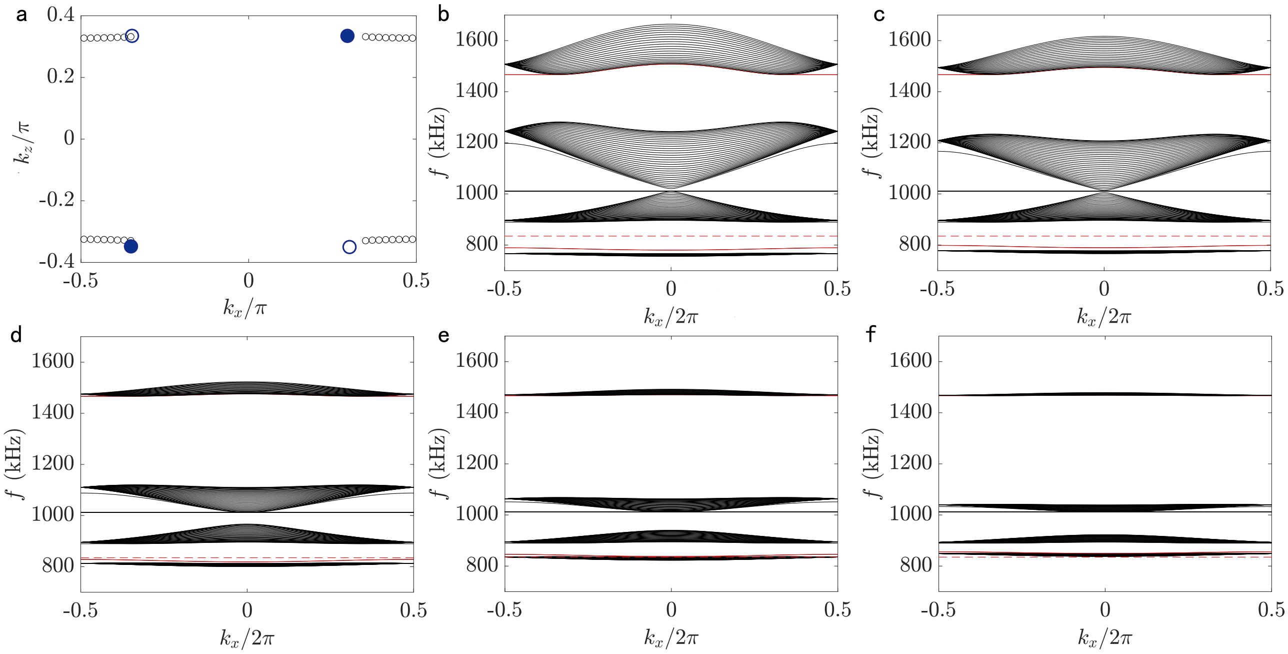

Supplementary Figure 5. **Fermi arc and dispersions of the slab geometry in frequency space.** **a** The contour of the surface states at the admittance of the Weyl points ($f = 835$ kHZ). The open and solid circles denote the Weyl points with opposite topological charges. **b-f** Dispersions of the slab geometry along the k_x direction for different k_z (1.56, 1.66, 1.975, 2.19, and 2.35). The solid red line denotes the surface state dispersion and the dashed line shows the position of $f = 835$ kHZ.

The homogeneous equations of motion ($\mathbf{I} = 0$) can be rewritten as $2N$ differential equations of first order [1]:

$$-i\frac{d}{dt}\psi(t) = \mathcal{H}_S\psi(t), \tag{14}$$

with $\psi = (\dot{\mathbf{V}}(t), \mathbf{V}(t))^T$ and the Hamiltonian block matrix being $\mathcal{H}_S = i \begin{pmatrix} 0 & C^{-1}L \\ -\mathbf{1} & 0 \end{pmatrix}$. By diagonalizing $\mathcal{H}_S$, we can obtain the frequency dispersion $\omega(k_x)$.

[1] Hofmann, T., Helbig, T., Lee, C. H., Greiter, M. & Thomale, R. Chiral Voltage Propagation and Calibration in a Topolectrical Chern Circuit, Phys. Rev. Lett. **122**, 247702 (2019).

[2] Jung, M., Yu, Y. & Shvets, G. Exact higher-order bulk-boundary correspondence of corner-localized states, Phys. Rev. B **104**, 195437 (2021).

[3] van Miert, G. & Ortix, C. On the topological immunity of corner states in two-dimensional crystalline insulators, npj Quantum Mater. **5**, 63 (2020).
